# Efficacy of Adjunctive Therapy with Zonisamide Versus Increased Dose of Levodopa for Motor Symptoms in Patients with Dementia with Lewy Bodies: The Randomized, Controlled, Non-Inferiority DUEL Study

**DOI:** 10.3233/JAD-230335

**Published:** 2023-08-29

**Authors:** Manabu Ikeda, Etsuro Mori, Satoshi Orimo, Tomomi Yamada, Osamu Konishi

**Affiliations:** a Department of Psychiatry, Course of Integrated Medicine, Graduate School of Medicine, Osaka University, Osaka, Japan; b Department of Behavioral Neurology and Neuropsychiatry, United Graduate School of Child Development, Osaka University, Osaka, Japan; c Department of Neurology, Kamiyoga Setagaya Street Clinic, Tokyo, Japan; d Department of Medical Innovation, Osaka University Hospital, Osaka, Japan; e Medical Science, Sumitomo Pharma Co., Ltd., Osaka, Japan

**Keywords:** Alzheimer’s disease, dementia with Lewy bodies, levodopa, Lewy body disease, parkinsonism, randomized controlled trial, zonisamide

## Abstract

**Background:**

In patients with dementia with Lewy bodies (DLB), it is unknown whether adjunct zonisamide is as effective and safe as increasing levodopa dose when levodopa has inadequate efficacy on parkinsonism.

**Objective:**

To compare adjunct zonisamide 25 mg/day versus an increased levodopa dose (increased by 100 mg/day) in patients with DLB treated with levodopa ≤300 mg/day for parkinsonism.

**Methods:**

The DUEL study was a multicenter, randomized, controlled, open-label, parallel-group, interventional, non-inferiority trial. During the observation period, levodopa was administered at ≤300 mg/day for 4 weeks. Subsequently, patients were randomized to receive adjunct zonisamide 25 mg/day or levodopa increased by 100 mg/day.

**Results:**

Respective adjusted mean changes in MDS-UPDRS Part III total score at 16 and 24 weeks (primary endpoint) were –6.3 and –4.4 in the zonisamide add-on and –0.8 and 2.0 in the levodopa increase groups. The adjusted mean difference at 24 weeks was –6.4 (95% confidence interval [CI] –13.5, 0.7); the upper limit of the 95% CI (0.7) was lower than the non-inferiority margin (3.0). No significant between-group differences were observed in total scores of the MDS-UPDRS Part II, Eating Questionnaire, EuroQol-5 dimension-5 level, Zarit Caregiver Burden Interview, or other secondary endpoints. No notable between-group differences were observed in adverse event incidences.

**Conclusion:**

Adjunct zonisamide 25 mg/day may yield moderate improvement in motor symptoms in patients with DLB when the levodopa effect is insufficient, but it could not be verified that the zonisamide 25 mg/day was as effective as levodopa 100 mg/day because levodopa showed no sufficient efficacy as assumed.

## INTRODUCTION

Along with Alzheimer’s disease and vascular dementia, dementia with Lewy bodies (DLB) is one of the three most common forms of dementia, accounting for approximately 10% –20% of dementia cases [1, 2]. DLB is characterized by progressive cognitive impairment with various clinical features, such as fluctuating cognition, visual hallucinations, rapid eye movement sleep behavior disorder, and parkinsonism. Other clinical features include depression, autonomic dysfunction (i.e., constipation, orthostatic hypotension, and urination disorder), severe neuroleptic hypersensitivity, delusions, and hallucinations other than visual ones [3].

Abundant Lewy bodies composed of alpha-synuclein in the cerebral cortex, midbrain, or peripheral autonomic nervous system of patients with DLB and Parkinson’s disease (PD) dementia (PDD) indicate that both disorders are part of the same disease spectrum and present similar pathological features [4, 5]. A degenerative loss of dopamine innervation in the substantia nigra striatum is thought to be one of the causes of parkinsonism in DLB [6]. There is no curative treatment for DLB, and current pharmacotherapy focuses on symptom management. Available medications modulate neurotransmitter functions to treat cognitive dysfunction, behavioral and psychological symptoms of dementia (BPSD), and parkinsonism [7].

A survey of patients with DLB revealed that parkinsonism is one of the most troubling symptoms in daily life and that this treatment need remains unmet [8]. Conversely, an Internet survey of psychiatrists who treat patients with DLB reported that treatment priority for parkinsonism is low; thus, this patient– physician perception gap is an urgent problem [9]. It has also been reported that, rather than treatment effectiveness, preventing the worsening of hallucinations and delusions may be the most important consideration in treating patients with parkinsonism in DLB [10].

According to the Clinical Practice Guideline for Dementia 2017 by the Japanese Society of Neurology [11], levodopa is recommended for treating parkinsonism accompanied by DLB, per pharmacotherapy for PD. The guideline recommends starting levodopa at low doses and increasing gradually to the lowest necessary dose to avoid/reduce adverse events (AEs). Although levodopa is effective for parkinsonism in DLB, treatment response tends to be lower than that in PDD [12]. Increasing the levodopa dose may also worsen BPSD and cause delirium [13, 14]. The Internet survey of physicians treating DLB suggested that safety is the most important factor in treating DLB, and a lower proportion of psychiatrists (i.e., who are not specialists in treating parkinsonism) than neurologists selected levodopa/decarboxylase inhibitors as initial treatment [10]. This may be because of inadequate evidence of efficacy and safety in treating DLB parkinsonism.

Zonisamide, originally an antiepileptic drug, has several mechanisms of action to improve motor dysfunction associated with PD and DLB, such as dopaminergic and nondopaminergic mechanisms, including inhibition of monoamine oxidase B activity, inhibition of T-type Ca^2 +^ channels, and inhibition of Na^+^ channels [15–17]. Zonisamide was approved in Japan for treating patients with PD in 2009 and patients with parkinsonism in DLB in 2018 [18]. Reportedly, when used adjunctively with levodopa, zonisamide 25 mg/day improves motor symptoms of PD [19] and DLB without exacerbating psychiatric symptoms [20, 21]. Although the efficacy for parkinsonism and safety of adjunctive zonisamide 25 mg/day and of placebo have been compared in previous clinical trials in patients with DLB, it is unknown whether the addition of zonisamide is as effective and safe as increasing levodopa dose when levodopa is administered for parkinsonism but does not reach adequate efficacy. Thus, the DUEL study aimed to clarify the effects of zonisamide 25 mg/day on parkinsonism versus an increased levodopa dose (dose increased by 100 mg/day) in patients with parkinsonism in DLB who were treated with levodopa ≤300 mg/day.

## MATERIALS AND METHODS

### Study design

The DUEL study was a multicenter, randomized, controlled, open-label, parallel-group, interventional, non-inferiority trial conducted in 47 centers in Japan between March 2021 and July 2022. It comprised a 4-week observation period and a 24-week evaluation period (Supplementary Figure 1). The evaluation period started on Day 1 (baseline) when the study drug was administered. After the observation period, patients were randomly assigned to the zonisamide add-on group or the levodopa increase group in a 1:1 ratio using the stratified block randomization method based on The Movement Disorder Society-Sponsored Revision of the Unified Parkinson’s Disease Rating Scale (MDS-UPDRS) Part III total score (<31 or ≥31) at baseline as an allocation adjustment factor via the Interactive Web Response System. The time point after randomization and before the evaluation period (Visit 2, Day –1) was defined as baseline. As this was an open-label study, no blinding procedures were performed.

The study received approval from the Osaka University Clinical Research Review Committee (CRB No. CRB5180007), which notified all participating centers of the approval per the Clinical Research Act in Japan. The CRB conducted a central review, and after CRB approval, the investigator of each site obtained permission from their administrator to start the research. The study was conducted according to the ethical principles, clinical research laws, and relevant notifications stipulated in the Declaration of Helsinki (as revised in 2013). This study was registered in the Japan Registry of Clinical Trials under the identifier jRCTs051200054. The investigators explained the study procedures to patients considered suitable for enrollment and their caregivers or alternative representatives, who provided written informed consent once they understood the study procedures and agreed to participate.

### Patients

The study included patients with DLB who had residual parkinsonism on doses of levodopa of ≤300 mg/day and met all the following eligibility criteria at the beginning of the observation period and before the beginning of the evaluation period.

The inclusion criteria were as follows: outpatients aged 50 to <90 years; who met the diagnostic criteria for probable DLB with symptoms of parkinsonism as core symptoms [3]; with MDS-UPDRS Part III total score of ≥20; with a history of hallucinations or delusions; with a caregiver who provided informed consent and who was able to provide patient information, to manage patients’ medication adherence, and to accompany the patient on hospital visits throughout the study period; who had been taking levodopa ≤300 mg/day at a constant dosage and administration for >4 weeks before Day 1; who had been taking a constant antidementia drugs dosage for >4 weeks before Day 1 if using medications for the treatment of dementia; and who were receiving a constant dosage of restricted concomitant medications for >2 weeks before Day 1.

The main exclusion criteria were as follows: patients with parkinsonism due to causes other than DLB; patients with PDD (according to the 1-year rule [3]); with total Mini-Mental State Examination (MMSE) scores of <10; with epilepsy; receiving antiparkinsonian medications other than levodopa (including carbidopa/levodopa/entacapone [Stalevo^®^]), droxidopa, benzamide antipsychotics (sulpiride, sultopride, tiapride, and nemonapride), antipsychotics other than quetiapine, metoclopramide, and nicergoline; previously receiving zonisamide; having undergone surgery for parkinsonism, such as stereotactic brain surgery; and judged by the physician to be inappropriate for the study participation because of severe psychiatric symptoms, such as confusion, hallucinations, delusions, or abnormal behavior.

### Intervention

During the observation period, levodopa/decarboxylase inhibitors were administered at ≤300 mg/day for 4 consecutive weeks, maintaining constant dosage and administration. This baseline treatment with levodopa was also maintained during the evaluation period at the same dosage and administration as during observation (Supplementary Figure 1).

At the end of the observation period, subjects were randomized to receive either zonisamide 25 mg/day in addition to levodopa (zonisamide add-on group) or an increased levodopa dose of 100 mg/day (100 mg once daily or 50 mg twice daily at the physician’s discretion) (levodopa increase group), and the timing of the administration was set at the physician’s discretion. Therefore, although this was an open-label study, the method of administration of the increased levodopa dose of 100 mg differed among patients. During the evaluation period, the study drug (zonisamide or levodopa) was administered orally for 24 weeks (zonisamide 25-mg tablets once daily in the morning). The final observation was made at the end of the study drug administration. Restricted concomitant medications included antihypertensive, central nervous system, cardiovascular, and gastrointestinal disorder drugs, and Chinese herbal preparations.

### Study endpoints

The primary endpoint was the change in MDS-UPDRS Part III total score from baseline at 24 weeks. The secondary endpoints included the total score and sub-items in MDS-UPDRS Part II and III [22], Neuropsychiatric Inventory (NPI)-12, NPI-10 [23], MMSE [24], the Japanese version of the Rapid Eye Movement Sleep Behavior Disorder Questionnaire (RBDQ-JP) [25], Eating Questionnaire [26], EuroQol 5 dimensions 5-level (EQ-5D-5L) [27], Zarit Caregiver Burden Interview (ZBI) [28], and Fall Questionnaire during the evaluation period. The Fall Questionnaire we had originally developed asked patients and/or their caregivers to rate the frequency of falls (0 = never; 1 = occasionally, less than once per week; 2 = often, about once per week; 3 = frequently, several times per week but less than every day; 4 = very frequently, once or more per day or continuously). Regarding the MDS-UPDRS Part III total score, the percentages of responders who improved by ≥10%, ≥20%, and ≥30% from baseline were also calculated. Other secondary endpoints were the number of correct and illusory responses and detection misses in the pareidolia test [29]; the risk of fall, presence of a fracture, or surgical treatment for fall injuries; and event occurrence of time to first fall and first fracture, study discontinuation and discontinuation due to worsening BPSD, and treatment-emergent AEs (TEAEs) and adverse drug reactions (ADRs).

Efficacy and safety endpoints, except for AEs and ADRs, were evaluated by a third party (e.g., physician or clinical psychologist, physiotherapist) other than the principal investigator in charge of each patient. The MDS-UPDRS Part III was evaluated by a third party (e.g., physician, clinical psychologist, physical therapist) who had been certified by the e-learning course developed by the Movement Disorder Society.

### Sample size calculation and non-inferiority margin

In previous clinical studies of patients with DLB [20, 21, 30], the mean change in UPDRS Part III total score in the zonisamide 25 mg/day add-on group ranged from 4.2 to 5.6 with a standard deviation (SD) of 6.2 to 7.2. Because the treatment durations of this and previous studies were considered (to emphasize the results during long-term treatment), the mean and SD of the UPDRS Part III total score change were estimated at 5.6 and 7.0, respectively.

In three clinical studies of levodopa in patients with parkinsonism in DLB [12, 14, 31], the mean change in UPDRS Part III total score for the levodopa groups ranged from 3.7 to 6.4. Based on these results, we assumed that the change in the UPDRS Part III total score would be 4.4 for an increased levodopa dose of 100 mg/day. The UPDRS Part III total score was converted to the MDS-UPDRS Part III total score using the aforementioned calibration formula of Goetz et al. [32].

For the primary endpoint (change from baseline in MDS-UPDRS Part III total score at 24 weeks), the mean values were assumed to be 6.72 and 5.28 for the zonisamide add-on and levodopa increase groups, respectively, with a common SD of 8.40 for both groups. Regarding the non-inferiority margin, a previous study reported that a clinically important difference in the UPDRS Part III total score change was 2.5 in patients with PD [33], which was the non-inferiority margin used in two recent non-inferiority trials in Japanese PD patients [34, 35]. Thus, we set the non-inferiority margin for this study at 2.5 of the UPDRS Part III total score change. Using the aforementioned calibration formula of Goetz et al. [32], the non-inferiority margin was set to 3.0 of the change in the MDS-UPDRS Part III total score. Under the above assumption, 58 patients per group (116 patients in total) were required to demonstrate the non-inferiority of zonisamide to levodopa with a power of 80% by a t-test with a one-sided significance level of 0.025. Assuming a dropout rate of 10%, the target sample size was set to 65 patients per group (130 patients in total for the two groups).

### Statistical analysis

The modified intention-to-treat (mITT) population was defined as all randomized patients who received at least one dose of the study drug and with available data on MDS-UPDRS Part III total score at baseline and after receiving study drugs. The per-protocol (PP) population was defined as all patients in the mITT population who complied with the study protocol. The safety population was defined as all patients enrolled in the study who received at least one dose of the study drug.

The primary analysis for efficacy was performed in the PP population, and the secondary analysis for efficacy was conducted in the mITT population. To analyze the primary endpoint, a mixed model for repeated measures (MMRM) was used to estimate the difference in the adjusted means between the two groups, its 95% confidence interval (CI), and p-value. The MMRM included the change from baseline in MDS-UPDRS Part III total score at 8, 16, and 24 weeks in the PP population as a response variable, treatment group, evaluation time point (week), and treatment group– week interactions as fixed effects, and the value of MDS-UPDRS Part III total score at baseline as a covariate. In the MMRM analysis, an unstructured covariance was assumed for the intrasubject errors at the three time points. Non-inferiority was demonstrated when the upper limit of the two-sided 95% CI was ≤3.0 for the adjusted mean difference between the two groups in the change of MDS-UPDRS Part III total score from baseline at 24 weeks. Furthermore, if non-inferiority was demonstrated, superiority was demonstrated when the upper limit of the 95% CI was less than 0. A similar analysis to the primary analysis was performed for the change in MDS-UPDRS Part III total score from baseline to 8 and 16 weeks in the PP population. The same statistical analysis method was used for the secondary analysis of the primary endpoint in the mITT population.

The secondary endpoints were analyzed in the mITT population. Regarding MDS-UPDRS Part II, RBDQ-JP, Eating Questionnaire, EQ-5D-5L, ZBI, Fall Questionnaire, NPI, MMSE, and pareidolia test, the statistical significance differences were tested by Wilcoxon signed-rank sum test for change from baseline and by Wilcoxon rank-sum test for intergroup differences. Time to first fall and first fracture, incidences of study discontinuation, and discontinuation due to worsening BPSD symptoms were estimated using the Kaplan– Meier Method, and these intergroup differences were tested by log-rank test. Intergroup differences in the percentages of responders in MDS-UPDRS Part III, proportion of patients with fall-related injuries, including fractures, due to falls at each time point, and proportions of patients undergoing surgical treatment for fall injuries were tested by Fisher’s exact test.

AEs were coded using the Medical Dictionary for Regulatory Activities (MedDRAversion 24.1). The number and percentage of patients in each treatment group who reported a TEAE and ADR were calculated and summarized by System Organ Class and Preferred Term.

A *p* value less than 0.05 was considered to be statistically significant. A closed testing procedure was used to adjust for the multiplicity of the non-inferiority and superiority hypotheses. The analysis was exploratory, and no other multiplicity adjustment was made for multiple endpoints or time points. All analyses were performed with SAS Version 9.4 or higher (SAS Institute Inc., Cary, NC, USA).

## RESULTS

### Patient disposition and characteristics

**Fig. 1:**
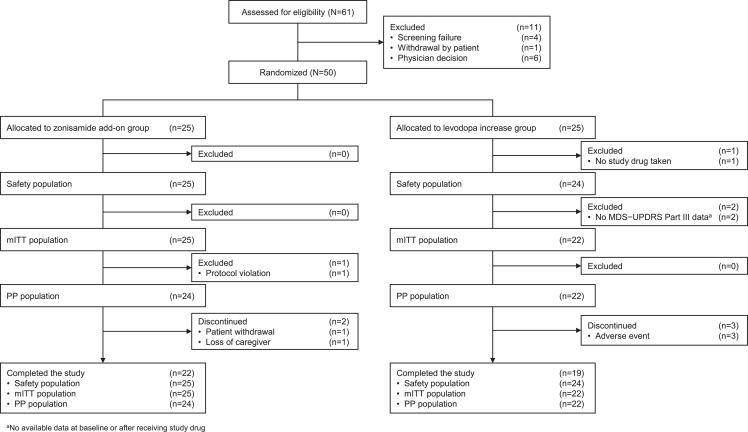
Patient disposition. MDS-UPDRS, The Movement Disorder Society-Sponsored Revision of the Unified Parkinson’s Disease Rating Scale; mITT, modified intention-to-treat; PP, per protocol.

From March 2021 to December 2021, 61 patients were enrolled, and 11 dropped out during the observation period. The remaining 50 patients were randomly assigned to the zonisamide add-on group (*n* = 25) or the levodopa increase group (*n* = 25). The safety, mITT, and PP populations included 25, 25, and 24 in the zonisamide add-on group and 24, 22, and 22 in the levodopa increase group, respectively (Fig. 1).

Table 1 summarizes the baseline demographic and clinical characteristics of patients. In the zonisamide add-on and levodopa increase groups, male patients comprised 52.0% and 45.5%; mean age (SD) was 79.5 (7.6) and 78.6 (6.9) years; DLB disease duration was 1.8 (1.3) and 1.6 (1.7) years; mean baseline scores for MDS-UPDRS Part III total score were 42.8 (15.9) and 37.3 (14.7); and mean levodopa doses were 210.0 (84.2) and 186.4 (86.2) mg/day, respectively. Over 70% of patients in each group were treated in the neurology department. No notable differences were observed in background factors between the two groups.

**Table 1:** Patient characteristics at baseline (mITT population)

	Zonisamide add-on group	Levodopa increase group	Total
	*n* = 25	*n* = 22	*N* = 47
Sex, male	13 (52.0)	10 (45.5)	23 (48.9)
Age, y, mean (SD)	79.5 (7.6)	78.6 (6.9)	79.1 (7.2)
Min–max	58–88	69–89	58–89
Specialty			
Psychiatry	5 (20.0)	4 (18.2)	9 (19.1)
Neurology	20 (80.0)	16 (72.7)	36 (76.6)
Other	0 (0.0)	2 (9.1)	2 (4.3)
Duration of DLB, y, mean (SD)	1.8 (1.3)	1.6 (1.7)	1.7 (1.5)
Duration of dementia, y, mean (SD)	4.1 (2.7)	3.5 (2.3)	3.8 (2.5)
Duration of parkinsonism, y, mean (SD)	2.4 (1.7)	2.4 (2.1)	2.4 (1.9)
Anti-dementia drug	20 (80.0)	20 (90.9)	40 (85.1)
Quetiapine	0 (0.0)	3 (13.6)	3 (6.4)
Yokukansan (herbal medicine)	4 (16.0)	1 (4.5)	5 (10.6)
MDS-UPDRS Part III total score, mean (SD)	42.8 (15.9)	37.3 (14.7)	40.2 (15.4)
Min–max	20–82	21–89	20–89
NPI-12 total score, mean (SD)	10.0 (9.3)	13.6 (13.1)	11.7 (11.3)
Min–max	0–27	1–45	0–45
MMSE total score, mean (SD)	21.7 (5.5)	21.4 (5.6)	21.6 (5.5)
Min–max	10–30	10–28	10–30
Levodopa dose, mg/day, mean (SD)	210.0 (84.2)	186.4 (86.2)	198.9 (85.0)
Min– max	50–300	50–300	5–300
≤100 mg	5 (20.0)	7 (31.8)	12 (25.5)
101–200 mg	10 (40.0)	9 (40.9)	19 (40.4)
201–300 mg	10 (40.0)	6 (27.3)	16 (34.0)

Data are *n* (%) or mean (SD). DLB, dementia with Lewy bodies; mITT, modified intention-to-treat; MDS-UPDRS, The Movement Disorder Society-Sponsored Revision of the Unified Parkinson’s Disease Rating Scale; MMSE, Mini-Mental State Examination; NPI, Neuropsychiatry Inventory; SD, standard deviation.

### Study endpoints

**Fig. 2:**
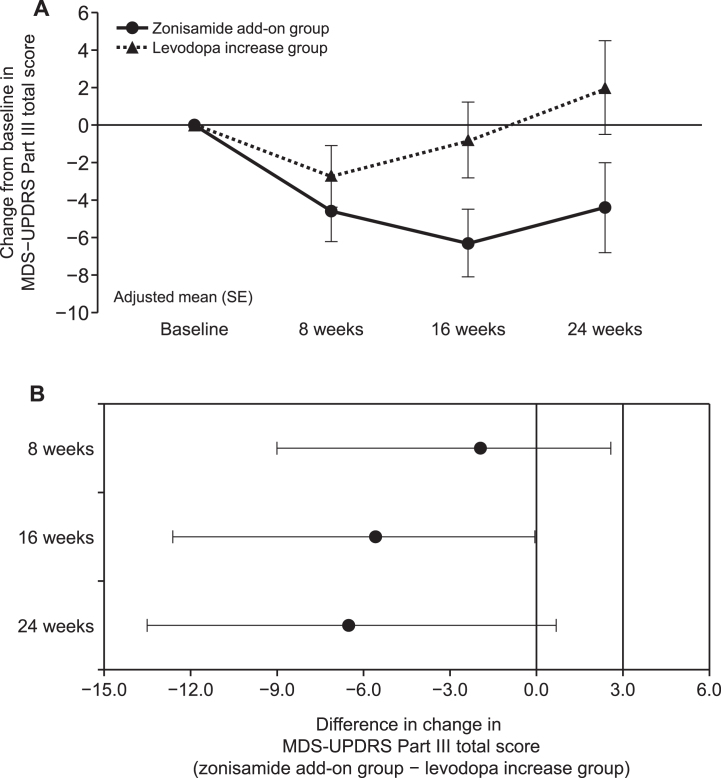
A) Change from baseline in the MDS-UPDRS Part III total score at each time point for the zonisamide add-on group (•) and the levodopa increase group (▴) (PP population, by MMRM). Each point represents the adjusted mean with SE. B) Difference in change in MDS-UPDRS Part III total score at each time point between the zonisamide add-on group and the levodopa increase group (PP population, by MMRM). Each point represents the adjusted mean with 95% CI. The upper limit ≤3.0 (non-inferiority margin) indicated non-inferiority of zonisamide add-on group and an upper limit ≤0 indicated superiority. CI, confidence interval; MDS-UPDRS, The Movement Disorder Society-Sponsored Revision of the Unified Parkinson’s Disease Rating Scale; MMRM, mixed model for repeated measure; PP, per-protocol; SE, standard error.

The adjusted mean (standard error) of the change from baseline in the MDS-UPDRS Part III total score at 24 weeks, the primary endpoint, was –4.4 (2.4) in the zonisamide add-on group and 2.0 (2.5) in the levodopa increase group (PP population) (Fig. 2A). Similar results were obtained in the mITT population (Supplementary Table 1). The adjusted mean difference between the two groups at 24 weeks was –6.4 (–13.5, 0.7). The upper limit of the 95% CI for the adjusted mean difference between the two groups at 24 weeks (0.7) was lower than the non-inferiority margin of 3.0, which was the criterion for non-inferiority of the zonisamide add-on group to the levodopa increase group (Fig. 2B). Conversely, no superiority was demonstrated at 24 weeks.

The change from baseline in the MDS-UPDRS Part III total score at 16 weeks was –6.3 (1.8) in the zonisamide add-on group and –0.8 (2.0) in the levodopa increase group (PP population). The adjusted mean difference at 16 weeks was –5.5 (–10.9, –0.05). The upper limit of the 95% CI (–0.05) was lower than 0, which was the criterion for superiority of zonisamide add-on to the levodopa dose increase (Fig. 2B).

**Fig. 3:**
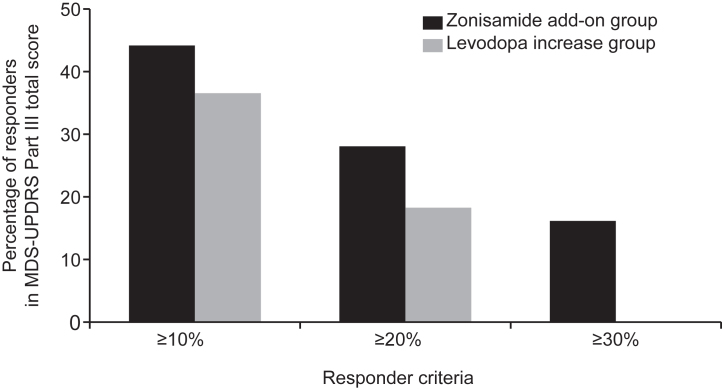
Percentage of responders in MDS-UPDRS Part III total score at 24 weeks (mITT population). MDS-UPDRS, The Movement Disorder Society-Sponsored Revision of the Unified Parkinson’s Disease Rating Scale; mITT, modified intention-to-treat.

The percentage of responders with ≥10%, ≥20%, and ≥30% change from baseline in the MDS-UPDRS Part III total score at 24 weeks was 44.0%, 28.0%, and 16.0% in the zonisamide add-on group and 36.4%, 18.2%, and 0% in the levodopa increase group (Fig. 3). There was no statistically significant difference between the two groups in the proportion of responders.

For the MDS-UPDRS Part III sub-items, patients in the zonisamide add-on group showed significant improvement in speech (*p* = 0.037), rigidity (*p* = 0.043), and pronation and supination of the hand (*p* = 0.014) vs those in the levodopa increase group at 24 weeks (Supplementary Table 2). For the other sub-items, there were no statistically significant differences between the two groups at 24 weeks.

The mean (SD) at baseline and the mean change from baseline at 24 weeks in the MDS-UPDRS Part II total score were 17.0 (8.3) and –1.5 (5.1) in the zonisamide add-on group and 13.8 (7.7) and –0.8 (3.8) in the levodopa increase group, respectively (Supplementary Table 3). A comparison of the mean change from baseline between the two groups in MDS-UPDRS Part II total score showed no statistically significant differences. Conversely, for MDS-UPDRS Part II sub-items, patients in the zonisamide add-on group showed significant improvement in saliva and drooling (*p* = 0.017), dressing (*p* = 0.030), and eating tasks (*p* = 0.043) vs those in the levodopa increase group at 24 weeks, which showed significant improvements in speech (*p* = 0.039).

There were no significant differences in the mean changes between the two groups in RBDQ-JP, Eating Questionnaire, EQ-5D-5L, or ZBI total scores at any time point (Supplementary Tables 4–7). Regarding the risk of falls, there were no statistically significant differences between the two groups in the mean change from baseline at any time point in the total falls score, frequency of falls, or requiring assistance to prevent falls (Supplementary Table 8), or in the number of fractures or surgical treatments for falling injuries (Supplementary Table 9).

Regarding the time from randomization to the first fall and first fracture (Supplementary Figure 2A, B), the rate of first fall was consistently higher in the zonisamide add-on group compared with the levodopa increase group until Day 150, at which point the rates were similar. The proportion of patients with first fractures remained low in both groups. In the levodopa increase group, no fractures occurred until Day 150. Regarding the time from randomization to discontinuation (Supplementary Figure 3A), the discontinuation rate remained low in both groups, and the trend remained similar between the two groups until the end of the study. The time from randomization to discontinuation for patients who required treatment for worsening BPSD could not be evaluated, as there were very few cases in either group (Supplementary Figure 3B).

### Safety

AEs were observed in 15 (60.0%) of 25 patients in the zonisamide add-on group and 14 (58.3%) of 24 patients in the levodopa increase group. One patient (4.0%) in the zonisamide add-on group had two ADRs (anxiety and urinary incontinence), and four patients (16.7%) in the levodopa increase group had one case each of decreased appetite, visual hallucinations, bipolar disorder, and right bundle branch block. No serious ADRs were observed in the zonisamide add-on group, and one serious ADR (bipolar disorder) was observed in the levodopa increase group. In the latter case, the patient had a medical history of bipolar disorder. As the episode of bipolar disorder (i.e., hypomanic symptoms) worsened after the levodopa dose was increased, the event was classified as an ADR. No ADRs leading to discontinuation of the study were observed in the zonisamide add-on group. ADRs leading to discontinuation were observed in three patients in the levodopa increase group (exacerbation of bipolar disorder, decreased appetite, and visual hallucinations).

Regarding the NPI-12 total score at 24 weeks (Supplementary Table 10), the levodopa increase group showed significantly lower scores than the zonisamide add-on group. For the MMSE total score at 24 weeks (Supplementary Table 11), there were no statistically significant differences in the mean change from baseline between the two groups. In the pareidolia test, no statistically significant differences were found in the mean change from baseline at 24 weeks between the two groups in the number of correct responses, illusory responses, and detection misses (Supplementary Table 12).

## DISCUSSION

This study aimed to demonstrate whether the efficacy of a zonisamide add-on dose of 25 mg/day on motor dysfunction was non-inferior to a 100 mg/day levodopa dose increase for 24 weeks in patients with DLB with a history of visual hallucinations or delusions who were receiving ≤300 mg/day of levodopa for parkinsonism. Although the target sample size was 130 patients, the analysis was conducted using data from 61 patients because of impaired recruitment as a result of the COVID-19 pandemic. In this study, the non-inferiority margin for the primary endpoint, adjusted mean change from baseline in the MDS-UPDRS Part III total score at 24 weeks, was reached in the zonisamide add-on group. There were no statistically significant differences between the two groups in the total scores of the secondary endpoints. Patients in the zonisamide add-on group showed significant improvement in specific MDS-UPDRS Part II sub-items, including saliva and drooling, dressing, and eating tasks at 24 weeks compared with the levodopa increase group. No worsening of MMSE, NPI-12 scores, cognitive function, or BPSD was observed in either group. Additionally, no notable differences were observed in the incidences of AEs between the two groups. The number of ADRs, serious ADRs, and ADRs leading to discontinuation in the zonisamide add-on group tended to be smaller than in the levodopa increase group.

The adjusted mean difference between the two groups of the MDS-UPDRS Part III total score at 24 weeks in the PP population was –6.4, and the upper limit of the 95% CI (0.7) was lower than the non-inferiority margin of 3.0. Based on this result, the zonisamide add-on dose was non-inferior to the levodopa dose increase. The adjusted mean changes from the baseline MDS-UPDRS Part III total score at 16 and 24 weeks in the zonisamide add-on group (–6.3 and –4.4) were similar to previous reports, with a decrease in treatment effect from 16 to 24 weeks [21, 30, 36]. However, the percentage of responders with ≥10% improvement at 24 weeks (44%) in this study was lower than previously reported [20, 21, 30, 36, 37], possibly because of zonisamide being less effective at 24 weeks than at 16 weeks (maximum effect), and the patient population having more advanced disease (i.e., 5 points higher than the baseline MDS-UPDRS Part III total scores).

No appropriate studies have compared levodopa escalation with another adjunctive antiparkinsonian drug or placebo; thus, historical comparisons with previous studies are limited because of differences in study design and patient population. The response rate to an increased dose of 100 mg/day levodopa for motor symptoms was similar to previous studies [14], but other studies have reported greater improvement in the UPDRS Part III total score at 6 months [12, 31]. It is possible that the lack of improvement observed in the levodopa increase group in our study compared with previous studies was attributable to a higher baseline levodopa dose in previous studies or differences in patient populations. A study of a levodopa challenge test (250-mg dose) in 20 patients with DLB newly started on levodopa treatment yielded a responder rate of 55% [12]. In a study in which 19 patients with parkinsonism in DLB had already been treated with levodopa 100 mg/day, approximately 31.5% of patients improved by >10% from baseline in UPDRS Part III total score after an average of approximately 3 months of treatment [14]. A similar responder rate (36%) was obtained in this study, despite different treatment durations of 12 and 24 weeks.

There have been no reports on the minimal clinically important difference (MCID) for changes in parkinsonism in patients with DLB. However, a study of 653 patients with PD reported that the estimated minimal, moderate, and large clinically relevant differences in UPDRS Part III total score (MDS-UPDRS Part III total score equivalent) were –2.5, –5.2, and –10.8, respectively [33]. Other studies, albeit not in patients with DLB, have reported MCIDs of –3.25 points for detecting minimal but clinically pertinent improvement and 4.63 points for observing minimal but clinically pertinent worsening on the MDS-UPDRS Part III [38]. In this study, the maximum mean changes in the MDS-UPDRS Part III total score in the zonisamide add-on and levodopa increase groups were –6.3 and –2.7, and the difference in the mean change in total MDS-UPDRS Part III score between groups at 24 weeks was –6.4; these differences were within the MCID ranges mentioned above. Thus, zonisamide might significantly improve motor symptoms in patients with DLB.

Comparing this study with previous studies of zonisamide in patients with DLB developing parkinsonism [21], the incidence of AEs tended to be slightly higher. However, adding zonisamide to treatment for patients with DLB and parkinsonism did not increase incidence of ADRs, with no ADRs leading to patient discontinuation from the study. In comparison, the group receiving an increase in levodopa had a higher incidence of ADRs and ADRs leading to discontinuation (16.7% and 8.3%, respectively). Although few studies have reported on the incidence of ADRs with levodopa (10.5% to 16.7% with worsening visual hallucinations and psychiatric or other symptoms), the incidence of ADRs seems to be lower in this study than in the previous studies [12, 14, 31]. As this study comprised patients with DLB being treated with levodopa, the difference in dropout rates could be attributed to a greater proportion of patients who were highly tolerant to levodopa in this study than in previous studies. Ultimately, the incidences of ADRs and ADRs leading to discontinuation reported in this study were lower in the zonisamide add-on group than in the levodopa increase group. Overall, adding zonisamide to levodopa did not appear to result in major tolerability problems and did not worsen psychiatric symptoms such as hallucinations and delusions, unlike in the levodopa increase group.

In patients with Lewy body disease (PDD and DLB), it has been reported that the total MMSE score changed by –1.3, the total NPI-12 score remained unchanged, and the UPDRS Part III total score (MDS-UPDRS equivalent) increased by 3.2 (3.8) during 6-month follow-up [39]. Neither the MMSE nor the NPI-12 total scores worsened in the zonisamide add-on group, similar to previous studies [21]. Thus, a zonisamide add-on dose of 25 mg/day is considered well tolerated in patients with DLB, regardless of whether they have a history of visual hallucinations or delusions. Similar to the zonisamide add-on group, in the levodopa increase group, both MMSE and NPI-12 total scores did not worsen in this study, comparable to a previous study [40].

It can be difficult to interpret speech results in MDS-UPDRS Part II and Part III, as there are discrepancies between them. Interestingly, speech in MDS-UPDRS Part III was improved in the zonisamide add-on group, whereas speech in MDS-UPDRS Part II was improved in the levodopa increase group. The reason for this difference is unclear. It is worth noting that the levodopa group had greater reductions in NPI depression and apathy scores compared with the zonisamide group. This suggests that mood improvements might play a role in the improvement in speech seen in the levodopa group in MDS-UPDRS Part II. Another possible explanation is that a type 1 error is likely to occur because no correction for repeated comparisons was made due to the exploratory analysis.

Another highly important finding was that zonisamide significantly improved the MDS-UPDRS Part II sub-items of saliva and drooling (*p* = 0.017), dressing (*p* = 0.030), and eating tasks (*p* = 0.043) vs the levodopa increase group. However, there was no significant improvement in cognitive function (MMSE), BPSD (NPI), and Eating Questionnaire in the zonisamide group compared with the levodopa increase group. These results suggest that a moderate improvement in motor symptoms may have directly contributed to the improvement in activities of daily living.

The zonisamide add-on group showed a numerical improvement in motor dysfunction, and the non-inferiority of zonisamide add-on to the levodopa dose increase was demonstrated by statistical testing. However, the levodopa increase group did not significantly reduce the MDS-UPDRS Part III total score at 24 weeks as expected. Therefore, the analytical sensitivity of the non-inferiority test (the ability to identify the test drug’s efficacy) was insufficient. There is another concern that an increase 100 mg/day of levodopa may have been too small to evaluate the equivalence/superiority of zonisamide. Further studies are required to verify non-inferiority or superiority.

This study has some limitations that should be considered when interpreting the findings. First, although a third party unaware of the study conducted the evaluations, it is impossible to completely rule out measurement bias because this was not a double-blind comparative study. Second, because of the COVID-19 pandemic, the planned number of subjects could not be enrolled, and hence the study was underpowered to detect the desired between-group differences. Finally, the efficacy of the levodopa increase could not be confirmed because the increase of levodopa as a control drug dose was limited to100 mg/day.

In conclusion, in patients with parkinsonism in DLB with a history of visual hallucinations or delusions treated with ≤300 mg/day of levodopa, a zonisamide add-on dose of 25 mg/day may be useful when the levodopa effect is insufficient. However, it cannot be clearly stated that effectiveness is verifiable from these results alone; a large, double-blind, comparative trial will be needed to verify the efficacy of this treatment approach.

## Supplementary Material

Supplementary Material

## Data Availability

The data supporting the findings of this study are available upon reasonable request from the corresponding author and Sumitomo Pharma Co., Ltd. The data are not publicly available due to privacy or ethical restrictions.
