# Supplementary material for: Efficacy of Adjunctive Therapy with Zonisamide Versus Increased Dose of Levodopa for Motor Symptoms in Patients with Dementia with Lewy Bodies: The Randomized, Controlled, Non-Inferiority DUEL Study
Source: J Alzheimers Dis. 2023 Aug 29;95(1):251–64. doi: 10.3233/JAD-230335 (PMC10578290; doi:10.3233/JAD-230335)
Supplement: Supplementary Material [file jad-95-jad230335-s001.pdf]

Supplementary Material

Efficacy of Adjunctive Therapy with Zonisamide Versus Increased Dose of Levodopa for Motor Symptoms in Patients with Dementia with Lewy Bodies: The Randomized, Controlled, Non-Inferiority DUEL Study

Supplementary Table 1. Change from baseline in MDS-UPDRS Part III at each time point (mITT population)

	**Zonisamide add-on group**			**Levodopa increase group**			p^a
	n	Mean (SD)	p^b	*n*	Mean (SD)	p^b	
8 weeks	23	−5.1 (8.5)	0.010	22	−2.1 (7.5)	0.015	0.452
16 weeks	23	−6.8 (10.8)	0.006	20	−0.3 (7.9)	0.312	0.083
24 weeks	22	−4.7 (9.3)	0.043	20	2.6 (14.3)	0.891	0.101

[a]Wilcoxon rank-sum test (zonisamide add-on group versus levodopa increase group)
[b]Wilcoxon signed-rank sum test (versus baseline)
MDS-UPDRS, The Movement Disorder Society-Sponsored Revision of the Unified Parkinson's Disease Rating Scale; mITT, modified intention to treat; SD, standard deviation.

Supplementary Table 2 Change from baseline in MDS-UPDRS Part III at 24 weeks (mITT population)

		Baseline		Change from baseline at 24 weeks				
		Zonisamide add-on group	Levodopa increase group	Zonisamide add-on group		Levodopa increase group		*p*[a]
		n = 25	*n* = 22	*n* = 22		*n* = 20		
		Mean (SD)	Mean (SD)	Mean (SD)	*p*[b]	Mean (SD)	*p*[b]	
3.1	Speech	1.2 (0.8)	1.1 (0.8)	−0.2 (0.5)	0.125	0.2 (0.7)	0.359	0.037
3.2	Facial expression	1.7 (0.8)	1.5 (0.9)	−0.1 (0.4)	0.625	0.2 (0.7)	0.508	0.164
3.3	Rigidity	8.0 (4.0)	7.7 (3.5)	−1.0 (2.0)	0.028	0.7 (3.6)	0.577	0.043
3.4	Finger tapping	3.6 (1.9)	3.0 (1.7)	−0.4 (1.6)	0.119	0.2 (1.3)	0.583	0.142
3.5	Hand movements	2.9 (1.9)	2.6 (1.6)	−0.1 (1.3)	0.741	0.1 (1.8)	0.917	0.835
3.6	Pronation-supination movement of hands	3.6 (1.8)	3.0 (1.5)	−0.4 (1.9)	0.378	0.8 (1.4)	0.045	0.014
3.7	Toe tapping	3.2 (2.1)	2.7 (1.5)	−0.5 (1.8)	0.285	0.2 (1.3)	0.712	0.329
3.8	Leg agility	2.9 (1.9)	2.4 (1.3)	−0.7 (1.6)	0.051	0.0 (1.7)	0.507	0.276
3.9	Arising from chair	1.2 (1.2)	1.0 (1.1)	0.0 (0.6)	1.000	0.2 (1.1)	0.828	0.619
3.10	Gait	1.6 (1.0)	1.4 (1.0)	−0.1 (0.4)	0.625	0.2 (0.8)	0.75	0.334
3.11	Freezing of gait	1.2 (1.1)	0.9 (1.2)	0.0 (0.5)	1.000	−0.1 (1.0)	0.797	0.577
3.12	Postural stability	2.0 (1.2)	1.8 (1.4)	−0.1 (0.8)	0.613	0.1 (1.5)	0.891	0.627
3.13	Posture	1.9 (1.0)	1.5 (0.9)	0.0 (0.8)	0.984	0.2 (0.8)	0.432	0.332
3.14	Global spontaneity of movement (body bradykinesia)	2.0 (1.0)	1.9 (0.9)	−0.1 (1.1)	0.687	−0.1 (0.8)	1.000	0.734
3.15	Postural tremor of the hands	1.3 (1.6)	1.3 (1.1)	0.1 (1.2)	0.823	0.3 (1.6)	0.439	0.915
3.16	Kinetic tremor of the hands	2.1 (1.8)	1.9 (1.8)	−0.5 (1.1)	0.031	−0.1 (1.2)	0.749	0.207
3.17	Rest tremor amplitude	1.6 (2.3)	1.1 (2.3)	0.0 (1.7)	0.719	0.0 (1.8)	0.656	0.627
3.18	Constancy of rest tremor	0.9 (1.2)	0.7 (1.1)	−0.2 (1.9)	0.500	−0.3 (0.8)	0.250	0.580
	Total	42.8 (15.9)	37.3 (14.7)	−4.7 (9.3)	0.043	2.6 (14.3)	0.891	0.101

[a]Wilcoxon rank-sum test (zonisamide add-on group versus levodopa increase group)

[b]Wilcoxon signed-rank sum test (versus baseline)

mITT, modified intention to treat; MDS-UPDRS, The Movement Disorder Society-Sponsored Revision of the Unified Parkinson's Disease Rating Scale; SD, standard deviation.

Supplementary Table 3. Change from baseline in MDS-UPDRS Part II at 24 weeks (mITT population)

		Baseline		Change from baseline at 24 weeks				
		Zonisamide add-on group	Levodopa increase group	Zonisamide add-on group		Levodopa increase group		*p*[a]
		n = 25	*n* = 22	*n* = 20		*n* = 18		
		Mean (SD)	Mean (SD)	Mean (SD)	*p*[b]	Mean (SD)	*p*[b]	
2.1	Speech	1.2 (1.3)	1.5 (1.5)	0.1 (1.2)	0.683	−0.8 (1.3)	0.031	0.039
2.2	Saliva and drooling	1.2 (1.1)	0.9 (1.2)	−0.4 (0.8)	0.055	0.3 (1.0)	0.344	0.017
2.3	Chewing and swallowing	1.1 (1.0)	0.5 (0.8)	−0.6 (1.1)	0.049	−0.1 (1.1)	0.625	0.170
2.4	Eating tasks	1.2 (0.7)	0.7 (0.9)	−0.3 (1.2)	0.454	0.4 (0.6)	0.031	0.043
2.5	Dressing	1.6 (1.0)	1.3 (1.0)	−0.5 (0.7)	0.020	0.1 (0.6)	1.000	0.030
2.6	Hygiene	1.0 (0.9)	1.0 (0.8)	0.2 (0.7)	0.508	−0.1 (0.8)	0.766	0.316
2.7	Handwriting	1.3 (1.1)	1.0 (0.9)	−0.1 (1.2)	0.784	0.3 (0.7)	0.188	0.292
2.8	Doing hobbies and other activities	1.4 (1.2)	1.8 (1.3)	0.2 (1.1)	0.421	−0.4 (1.2)	0.166	0.396
2.9	Turning in bed	0.9 (1.0)	0.7 (0.8)	−0.2 (1.0)	0.480	0.3 (1.0)	0.375	0.141
2.10	Tremor	1.2 (1.2)	0.8 (0.9)	−0.2 (0.9)	0.628	0.1 (0.5)	1.000	0.333
2.11	Getting out of bed, a car, or a deep chair	1.6 (1.2)	1.5 (1.1)	−0.1 (0.8)	1.000	−0.4 (0.9)	0.094	0.209
2.12	Walking and balance	1.8 (1.4)	1.5 (1.2)	0.1 (0.7)	0.766	−0.1 (1.1)	0.797	0.272
2.13	Freezing	1.3 (1.3)	0.8 (1.2)	0.2 (1.3)	0.709	−0.3 (0.8)	0.250	0.335
	Total	17.0 (8.3)	13.8 (7.7)	−1.5 (5.1)	0.162	−0.8 (3.8)	0.347	0.736

[a]Wilcoxon rank-sum test (zonisamide add-on group versus levodopa increase group)

[b]Wilcoxon signed-rank sum test (versus baseline)

mITT, modified intention to treat; MDS-UPDRS, The Movement Disorder Society-Sponsored Revision of the Unified Parkinson's Disease Rating Scale; SD, standard deviation.

Supplementary Table 4. Change from baseline in RBDQ-JP at 24 weeks (mITT population)

		Baseline				Change from baseline at 24 weeks						
		Zonisamide add-on group		Levodopa increase group		Zonisamide add-on group			Levodopa increase group			p^a
		Mean (SD)	*n*	Mean (SD)	*n*	Mean (SD)	*n*	p^b	Mean (SD)	*n*	p^b	
1	Frequent dreams	3.0 (2.1)	22	2.5 (1.8)	20	0.1 (2.0)	19	1.000	−0.9 (1.7)	16	0.071	0.204
2	Frequent nightmares	1.1 (1.6)	22	0.8 (1.3)	21	0.0 (1.8)	19	0.914	−0.1 (1.3)	17	0.938	0.800
3	Emotional dreams	0.9 (1.4)	22	0.5 (1.1)	22	0.4 (1.8)	18	0.352	0.5 (1.8)	15	0.328	0.682
4	Violent or aggressive dreams	0.9 (1.5)	22	1.0 (1.6)	21	−0.2 (1.6)	18	0.625	−0.5 (1.2)	17	0.125	0.548
5	Frightening dreams	0.5 (1.0)	22	1.2 (1.8)	20	0.3 (1.4)	18	0.453	−0.2 (1.9)	17	0.656	0.370
6	Sleep talking	6.0 (3.4)	23	5.7 (2.7)	22	0.2 (3.2)	18	0.741	−2.5 (2.9)	19	0.002	0.006
7	Shouting or yelling in sleep	3.5 (3.4)	23	2.7 (2.9)	22	−0.9 (3.0)	19	0.281	−0.4 (1.8)	19	0.500	0.778
8	Dream-related movements	3.1 (3.7)	21	1.9 (2.8)	21	0.2 (3.3)	17	0.947	−0.9 (2.8)	18	0.266	0.444
9	Falling out of bed	1.1 (1.7)	23	1.9 (3.1)	20	−0.5 (1.9)	19	0.375	−1.2 (3.2)	17	0.250	0.850
10	SRI	0.3 (0.7)	22	0.2 (0.9)	20	−0.1 (0.8)	19	1.000	−0.1 (1.2)	15	1.000	0.757
11	Attempts to assault/infare	0.3 (0.7)	22	0.4 (1.0)	21	−0.2 (0.6)	19	0.500	0.0 (1.3)	15	1.000	0.237
12	SRI related to dream content	0.4 (0.8)	22	0.2 (0.6)	21	−0.1 (0.5)	19	1.000	0.1 (0.9)	15	1.000	0.337
13	Disturbed sleep	1.1 (1.5)	22	1.1 (2.0)	20	−0.3 (1.2)	19	0.313	−0.4 (2.7)	15	0.578	1.000
	Total	22.0 (14.4)	21	19.9 (13.4)	20	−1.1 (10.0)	17	0.936	−7.1 (12.0)	15	0.020	0.075

[a]Wilcoxon rank-sum test (zonisamide add-on group versus levodopa increase group)

[b]Wilcoxon signed-rank sum test (versus baseline)

mITT, modified intention to treat; RBDQ-JP, Rapid Eye Movement Sleep Behavior Disorder Questionnaire Japanese version; SD, standard deviation, SRI, sleep-related injuries

Supplementary Table 5. Change from baseline in Eating Questionnaire at 24 weeks (mITT population)

			**Baseline**		**Change from baseline at 24 weeks**				
			Zonisamide add-on group	Levodopa increase group	Zonisamide add-on group		Levodopa increase group		p^a
			n = 25	n = 22	n = 22		n = 20		
			Mean (SD)	Mean (SD)	Mean (SD)	p^b	Mean (SD)	p^b	
1	Swallowing problems	Difficulty in swallowing food	0.7 (1.3)	0.5 (1.1)	0.8 (3.4)	0.502	0.4 (1.3)	0.266	0.685
2		Difficulty in swallowing liquids	0.4 (1.0)	0.8 (2.0)	0.1 (2.3)	1.000	−0.3 (1.7)	0.500	0.939
3		Coughing or choking when swallowing	1.4 (2.0)	1.1 (1.8)	0.1 (2.3)	0.838	−0.3 (1.2)	0.281	1.000
4		Taking a long time to swallow	1.6 (2.9)	0.7 (1.6)	0.4 (3.0)	0.672	0.1 (1.4)	0.844	0.586
5		Placing food in the mouth but not chewing it	0.5 (1.7)	0.1 (0.4)	0.5 (3.4)	0.750	−0.1 (0.5)	1.000	0.771
6		Chewing food but not swallowing it	0.2 (0.6)	0.0 (0.0)	0.2 (1.7)	1.000	0.2 (0.9)	1.000	0.671
7	Appetite change	Loss of appetite	0.8 (1.8)	0.4 (0.9)	−0.1 (1.5)	0.633	0.5 (2.9)	1.000	0.620
8		Increase in appetite	1.0 (2.7)	0.9 (2.0)	−0.8 (3.0)	0.328	−0.8 (1.9)	0.125	0.937
9		Seeking out food between meals	0.9 (1.9)	0.9 (1.3)	−0.3 (3.0)	0.719	−0.5 (1.6)	0.262	0.923
10		Overeating at mealtime	0.4 (1.3)	0.5 (1.1)	−0.3 (1.7)	0.625	−0.5 (1.1)	0.125	0.451
11		requesting more food	0.6 (1.8)	0.4 (1.1)	−0.6 (1.8)	0.188	−0.3 (0.9)	0.500	0.727
12		Reporting hunger	0.6 (1.2)	0.4 (0.8)	0.0 (1.2)	1.000	0.2 (0.7)	0.563	0.388
13		Reporting being overfull	1.1 (1.9)	0.4 (1.0)	0.2 (2.5)	1.000	0.4 (2.2)	0.625	0.312
14		Seeking out food between meals	0.0 (0.2)	0.0 (0.0)	0.0 (0.3)	1.000	0.0 (0.0)	−	1.000
15		Needs to limit food	0.3 (1.0)	0.3 (1.0)	0.3 (1.8)	1.000	0.2 (0.9)	1.000	0.592
16	Other eating behaviors	Suffering from sputum	2.2 (3.8)	0.3 (0.8)	−0.5 (3.1)	0.789	0.1 (1.1)	1.000	0.780
17		Suffering from fever	0.0 (0.0)	0.0 (0.0)	0.2 (0.7)	0.500	0.0 (0.0)	−	0.183
18		Fluctuation in appetite	0.2 (0.7)	0.4 (0.9)	0.1 (1.2)	0.844	−0.2 (1.2)	0.625	0.245

19	Fluctuation in swallowing ability	0.8 (2.2)	0.4 (0.7)	−0.1 (3.5)	0.703	0.0 (1.1)	0.781	0.890
20	Getting drowsy at mealtime	0.0 (0.0)	0.7 (2.1)	0.0 (0.0)	–	−0.5 (1.8)	0.500	0.142
21	Needs watching or help	1.6 (2.8)	0.4 (1.2)	0.3 (2.1)	0.813	0.2 (1.5)	0.875	0.613
22	Constipation	2.9 (3.5)	2.2 (3.4)	−0.9 (3.0)	0.207	−0.5 (3.5)	0.626	0.606
23	Total	18.2 (14.2)	11.8 (9.2)	−0.4 (24.9)	0.125	−2.0 (8.8)	0.307	0.596

[a]Wilcoxon rank-sum test (zonisamide add-on group versus levodopa increase group)

[b]Wilcoxon signed-rank sum test (versus baseline)

mITT, modified intention to treat; SD, standard deviation

Supplementary Table 6. Change from baseline in EQ-5D-5L (QOL score) at 24 weeks (mITT population)

	**Baseline**		**Change from baseline at 24 weeks**				p^a
	Zonisamide add-on group	Levodopa increase group	Zonisamide add-on group		Levodopa increase group		
	n = 25	*n* = 22	*n* = 23		*n* = 21		
	Mean (SD)	Mean (SD)	Mean (SD)	p^b	Mean (SD)	p^b	
QOL score	0.7068 (0.2123)	0.7450 (0.1346)	−0.0500 (0.2416)	0.695	0.0042 (0.1410)	0.442	0.613

[a]Wilcoxon rank-sum test (zonisamide add-on group versus levodopa increase group)

[b]Wilcoxon signed-rank sum test (versus baseline)

EQ-5D-5L, EuroQol 5 dimensions 5-level; mITT, modified intention to treat; QOL, quality of life; SD, standard deviation

Supplementary Table 7. Change from baseline in ZBI at 24 weeks (mITT population)

		Baseline		Change from baseline at 24 weeks				
		Zonisamide add-on group	Levodopa increase group	Zonisamide add-on group		Levodopa increase group		p^a
		n = 25	n = 22	n = 23		n = 22		
		Mean (SD)	Mean (SD)	Mean (SD)	p^b	Mean (SD)	p^b	
1	Do you feel that your relative asks for more help than he/she needs?	1.0 (1.3)	0.9 (1.1)	0.1 (0.8)	0.613	−0.3 (0.9)	0.172	0.049
2	Do you feel that because of the time you spend with your relative that you don't have enough time for yourself?	1.3 (1.3)	1.0 (0.9)	−0.2 (0.9)	0.391	−0.1 (1.2)	0.698	0.829
3	Do you feel stressed between caring for your relative and trying to meet other responsibilities for your family or work?	1.5 (1.4)	1.2 (0.9)	−0.3 (0.5)	0.063	−0.1 (0.7)	0.549	0.717
4	Do you feel embarrassed over your relative's behavior?	1.2 (1.2)	0.8 (0.9)	0.3 (1.2)	0.184	0.3 (0.8)	0.186	0.710
5	Do you feel angry when you are around your relative?	1.0 (1.2)	1.1 (0.8)	0.0 (1.2)	0.973	−0.1 (0.6)	0.727	0.568
6	Do you feel that your relative currently affects our relationships with other family members or friends in a negative way?	0.6 (0.9)	0.9 (1.1)	0.3 (0.9)	0.183	0.0 (1.0)	1.000	0.290
7	Are you afraid what the future holds for your relative?	1.9 (1.1)	2.1 (0.8)	−0.1 (1.2)	0.811	−0.6 (0.9)	0.009	0.043
8	Do you feel your relative is dependent on you?	2.2 (1.3)	2.6 (1.0)	0.1 (1.0)	0.577	−0.4 (1.0)	0.081	0.128
9	Do you feel strained when you are around your relative?	1.1 (1.3)	0.9 (0.9)	0.0 (1.3)	0.979	0.1 (1.0)	0.670	0.600
10	Do you feel your health has suffered because of your involvement with your relative?	0.9 (1.4)	0.2 (0.4)	−0.3 (1.1)	0.250	0.1 (0.8)	0.438	0.225

11	Do you feel that you do not have as much privacy as you would like because of your relative?	0.3 (0.6)	0.3 (0.7)	0.4 (1.2)	0.211	0.0 (0.4)	1.000	0.313
12	Do you feel that your social life has suffered because you are caring for your relative?	0.7 (1.0)	0.5 (0.8)	0.1 (1.2)	0.906	0.0 (0.8)	1.000	0.661
13	Do you feel uncomfortable about having friends over because of your relative?	0.2 (0.7)	0.2 (0.7)	0.4 (0.8)	0.063	0.5 (0.9)	0.031	0.809
14	Do you feel that your relative seems to expect you to take care of him/her as if you were the only one he/she could depend on?	1.8 (1.7)	2.0 (1.4)	0.0 (1.6)	0.873	0.0 (1.5)	0.813	0.784
15	Do you feel that you don’t have enough money to take care of your relative in addition to the rest of your expenses?	0.8 (1.2)	0.9 (1.2)	−0.2 (0.7)	0.359	−0.1 (0.7)	0.563	0.849
16	Do you feel that you will be unable to take care of your relative much longer?	0.9 (1.2)	0.5 (0.8)	0.1 (1.0)	0.672	0.3 (1.0)	0.305	0.634
17	Do you feel you have lost control of your life since your relative’s illness?	1.1 (1.3)	1.1 (0.8)	−0.1 (0.8)	0.813	0.0 (1.0)	1.000	0.661
18	Do you wish you could leave the care of your relative to someone else?	0.8 (0.9)	0.5 (0.6)	0.2 (0.7)	0.273	0.0 (0.7)	1.000	0.474
19	Do you feel uncertain about what to do about your relative?	0.8 (1.1)	0.8 (0.9)	0.3 (1.3)	0.292	0.2 (0.9)	0.354	0.873
20	Do you feel you should be doing more for your relative?	0.7 (1.2)	0.8 (1.0)	−0.4 (1.2)	0.188	0.3 (1.0)	0.221	0.073
21	Do you feel you could do a better job in caring for your relative?	0.7 (1.2)	0.4 (0.7)	−0.2 (0.7)	0.344	0.0 (1.0)	0.998	0.318
22	Overall, how burdened do you feel in caring for your relative?	1.4 (1.1)	1.7 (0.6)	0.0 (0.3)	1.000	−0.1 (0.9)	0.615	0.520
	Total	23.0 (18.0)	21.4 (8.5)	0.6 (12.5)	0.468	−0.1 (8.3)	0.949	0.481

[a]Wilcoxon rank-sum test (zonisamide add-on group versus levodopa increase group)
[b]Wilcoxon signed-rank sum test (versus baseline)

ZBI, Zarit Caregiver Burden Interview; mITT, modified intention to treat; SD, standard deviation

Supplementary Table 8. Change from baseline in Fall Questionnaire at 24 weeks (mITT population)

		**Baseline**		**Change from baseline at 24 weeks**				
		Zonisamide add-on group	Levodopa increase group	Zonisamide add-on group		Levodopa increase group		*p*[a]
		n = 25	*n* = 22	*n* = 22		*n* = 20		
		Mean (SD)	Mean (SD)	Mean (SD)	*p*[b]	Mean (SD)	*p*[b]	
1	Suffering from fall	0.5 (0.7)	0.2 (0.7)	−0.3 (0.6)	0.063	0.1 (0.8)	1.000	0.067
2	Needs watching to avoid falling	1.6 (1.8)	1.2 (1.6)	0.1 (1.5)	0.635	0.0 (1.1)	1.000	0.523
3	Needs help to avoid falling	1.3 (1.7)	0.9 (1.6)	0.0 (1.5)	0.995	−0.4 (1.8)	0.484	0.307
	Total	3.4 (3.7)	2.3 (3.3)	−0.2 (3.1)	0.890	−0.3 (2.7)	0.746	0.827

[a]Wilcoxon rank-sum test (zonisamide add-on group versus levodopa increase group)

[b]Wilcoxon signed-rank sum test (versus baseline)

mITT, modified intention to treat; SD, standard deviation

Supplementary Table 9. Proportion of patients with fall-related injuries, including fractures, due to falls at each time point, and proportions of patients undergoing surgical treatment for fall injuries (mITT population)

	Time	Category	Number of cases (%) Zonisamide add-on group $N = 25$	Levodopa increase group $N = 22$	p^{a}
Presence of injuries, including fractures due to falls	0 week (Visit 2)	*n*	25	22	
		Presence	2 (8.0)	0 (0.0)	0.491
		Absence	23 (92.0)	22 (100.0)	
	4 weeks (Visit 3)	*n*	24	21	
		Presence	2 (8.3)	0 (0.0)	0.491
		Absence	22 (91.7)	21 (100.0)	
	8 weeks (Visit 4)	*n*	25	22	
		Presence	1 (4.0)	0 (0.0)	1.000
		Absence	24 (96.0)	22 (100.0)	
	16 weeks (Visit 5)	*n*	23	20	
		Presence	0 (0.0)	0 (0.0)	
		Absence	23 (100.0)	20 (100.0)	
	24 weeks (Visit 6)	*n*	22	20	
		Presence	1 (4.5)	2 (10.0)	0.598
		Absence	21 (95.5)	18 (90.0)	
Surgical treatment for injuries caused by falls	0 week (Visit 2)	*n*	25	22	
		Presence	1 (4.0)	0 (0.0)	1.000
		Absence	24 (96.0)	22 (100.0)	
	4 weeks (Visit 3)	*n*	24	21	
		Presence	1 (4.2)	0 (0.0)	1.000
		Absence	23 (95.8)	21 (100.0)	
	8 weeks (Visit 4)	*n*	25	22	
		Presence	1 (4.0)	0 (0.0)	1.000
		Absence	24 (96.0)	22 (100.0)	
	16 weeks (Visit 5)	*n*	23	20	
		Presence	0 (0.0)	0 (0.0)	—
		Absence	23 (100.0)	20 (100.0)	
	24 weeks	*n*	22	20	

	(Visit 6)	Presence	1 (4.5)	2 (10.0)	0.598
		Absence	21 (95.5)	18 (90.0)	

[a]Fisher’s exact test (zonisamide add-on group versus levodopa increase group)

Data are n (%). mITT, modified intention to treat

Supplementary Table 10. Change from baseline in NPI at 24 weeks (Safety population)

		Baseline		Change from baseline at 24 weeks				
		Zonisamide add-on group	Levodopa increase group	Zonisamide add-on group		Levodopa increase group		*p*[a]
		n = 25	*n* = 24	*n* = 22		*n* = 20		
		Mean (SD)	Mean (SD)	Mean (SD)	*p*[b]	Mean (SD)	*p*[b]	
1	Delusions	0.0 (0.2)	1.1 (2.3)	1.0 (2.2)	0.063	−0.9 (2.5)	0.254	0.025
2	Hallucinations	1.3 (1.5)	1.7 (2.7)	0.0 (1.0)	0.984	−0.9 (1.9)	0.063	0.102
3	Agitation/Aggression	0.6 (1.0)	0.6 (1.3)	0.2 (2.0)	0.992	−0.2 (1.2)	1.000	0.764
4	Depression/Dysphoria	0.7 (1.1)	1.6 (2.4)	0.4 (1.9)	0.545	−0.8 (2.5)	0.255	0.430
5	Anxiety	0.7 (1.6)	1.0 (2.0)	0.0 (1.9)	0.859	−0.6 (1.5)	0.086	0.162
6	Elation/Euphoria	0.3 (1.0)	0.0 (0.0)	−0.3 (1.0)	0.500	0.0 (0.0)	–	0.183
7	Apathy/Indifference	2.2 (2.8)	2.9 (3.4)	−0.6 (2.6)	0.453	−1.0 (2.9)	0.207	0.851
8	Disinhibition	0.3 (1.0)	0.1 (0.4)	−0.2 (1.0)	0.375	−0.1 (0.5)	1.000	0.444
9	Irritability/Lability	1.4 (2.4)	0.4 (1.2)	−0.6 (1.2)	0.047	−0.2 (1.0)	0.750	0.225
10	Aberrant Motor Behavior	0.3 (0.9)	1.3 (2.3)	0.1 (1.6)	1.000	−1.0 (2.8)	0.156	0.107
11	Sleep	1.0 (2.6)	2.0 (3.1)	1.5 (3.2)	0.039	−0.8 (3.0)	0.332	0.057
12	Appetite and eating disorders	1.2 (2.9)	0.6 (1.2)	0.5 (1.8)	0.313	0.4 (2.0)	0.750	0.647
	NPI-10 total	7.8 (7.0)	10.8 (10.4)	0.0 (8.7)	0.730	−5.5 (0.5)	0.026	0.088
	NPI-12 total	10.0 (9.3)	13.4 (12.6)	2.0 (11.8)	0.620	−6.0(10.8)	0.031	0.035

[a]Wilcoxon rank-sum test (zonisamide add-on group versus levodopa increase group)
[b]Wilcoxon signed-rank sum test (versus baseline)
NPI, Neuropsychiatric Inventory; SD, standard deviation

Supplementary Table 11. Change from baseline in MMSE at 24 weeks (Safety population)

		Baseline		Change from baseline at 24 weeks				p^a
		Zonisamide add-on group	Levodopa increase group	Zonisamide add-on group		Levodopa increase group		
		$n = 25$	$n = 24$	$n = 22$		$n = 20$		
		Mean (SD)	Mean (SD)	Mean (SD)	p^b	Mean (SD)	p^b	
1	Orientation to time	3.4 (1.9)	3.5 (1.4)	−0.2 (1.2)	0.516	−0.5 (0.8)	0.033	0.169
2	Orientation to place	4.0 (1.6)	4.1 (1.1)	0.0 (1.1)	0.916	−0.6 (1.1)	0.028	0.060
3	Registration	2.9 (0.3)	2.7 (0.6)	−0.2 (0.6)	0.250	0.0 (0.8)	1.000	0.352
4	Attention and calculation	2.0 (1.5)	1.9 (1.6)	0.2 (1.3)	0.384	0.7 (1.9)	0.155	0.589
5	Recall	1.6 (1.3)	2.0 (1.1)	0.0 (0.8)	0.973	−0.3 (1.2)	0.309	0.638
6	Naming	2.0 (0.2)	2.0 (0.0)	0.0 (0.4)	1.000	0.0 (0.0)	–	0.592
7	Repetition	1.0 (0.0)	1.0 (0.2)	0.0 (0.0)	–	0.1 (0.2)	1.000	0.317
8	Comprehension	2.4 (0.8)	2.3 (1.0)	0.1 (0.9)	0.464	0.2 (0.5)	0.219	0.587
9	Reading	1.0 (0.0)	0.9 (0.3)	0.0 (0.2)	1.000	0.1 (0.2)	1.000	0.172
10	Writing	0.9 (0.3)	0.8 (0.4)	0.0 (0.4)	1.000	−0.3 (0.5)	0.031	0.044
11	Drawing	0.5 (0.5)	0.7 (0.5)	0.0 (0.6)	1.000	0.1 (0.4)	1.000	1.000
	Total	21.7 (5.5)	21.8 (5.6)	−0.2 (2.8)	0.990	−0.6 (3.7)	0.481	0.751

[a]Wilcoxon rank-sum test (zonisamide add-on group versus levodopa increase group)
[b]Wilcoxon signed-rank sum test (versus baseline)
MMSE, Mini-Mental State Examination; SD, standard deviation

Supplementary Table 12. Change from baseline in Pareidolia Test at 24 weeks (Safety population)

		Baseline		Change from baseline at 24 weeks				
		Zonisamide add-on group	Levodopa increase group	Zonisamide add-on group		Levodopa increase group		*p*[a]
		n = 25	*n* = 24	*n* = 22		*n* = 20		
		Mean (SD)	Mean (SD)	Mean (SD)	*p*[b]	Mean (SD)	*p*[b]	
1	Correct responses	33.2 (8.4)	31.5 (10.0)	1.6 (3.9)	0.051	0.8 (7.1)	0.435	0.790
2	Illusory responses	5.8 (7.9)	7.3 (9.3)	−1.6 (4.1)	0.060	−1.0 (6.9)	0.385	0.949
3	Detection misses	1.1 (1.7)	1.1 (1.3)	0.0 (1.1)	0.998	0.2 (1.7)	0.585	0.724

[a]Wilcoxon rank-sum test (zonisamide add-on group versus levodopa increase group)

[b]Wilcoxon signed-rank sum test (versus baseline)

Correct responses: subjects correctly responded “nothing exists” to the noise stimuli or correctly detected the embedded image in the images that contained faces.

Illusory responses: subjects falsely found faces in images without a face.

Detection misses: subjects did not detect the embedded faces. SD, standard deviation

Supplementary Figure 1. Study design

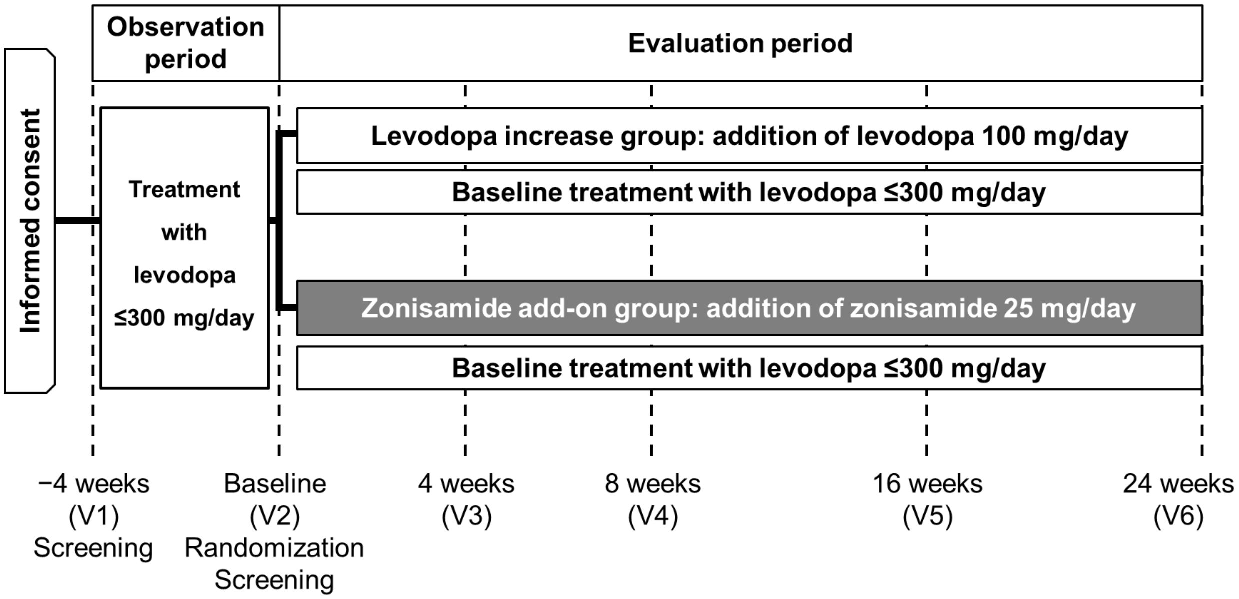

V, visit

Supplementary Figure 2. Kaplan–Meier plot for time from randomization to A) first fall and B) first fracture (mITT population)

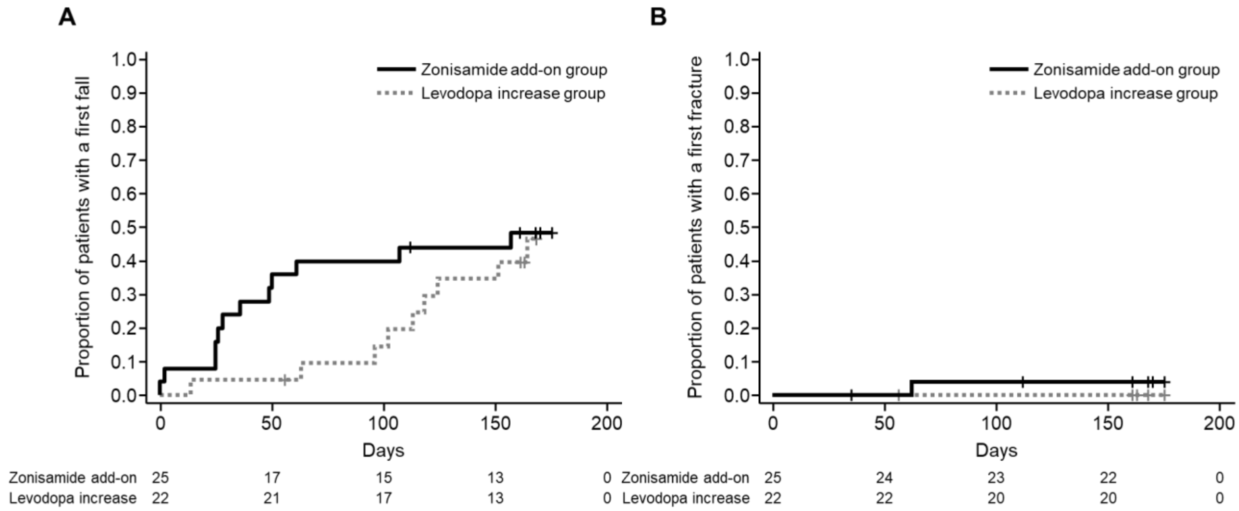

Log-rank $p = 0.444$ for first fall (zonisamide add-on group versus levodopa increase group)
Log-rank $p = 0.361$ for the first fracture (zonisamide add-on group versus levodopa increase group)
mITT, modified intention to treat; SD, standard deviation.

Supplementary Figure 3. Kaplan–Meier plot for time from randomization to A) discontinuation and B) to discontinuation of treatment for worsening BPSD symptoms

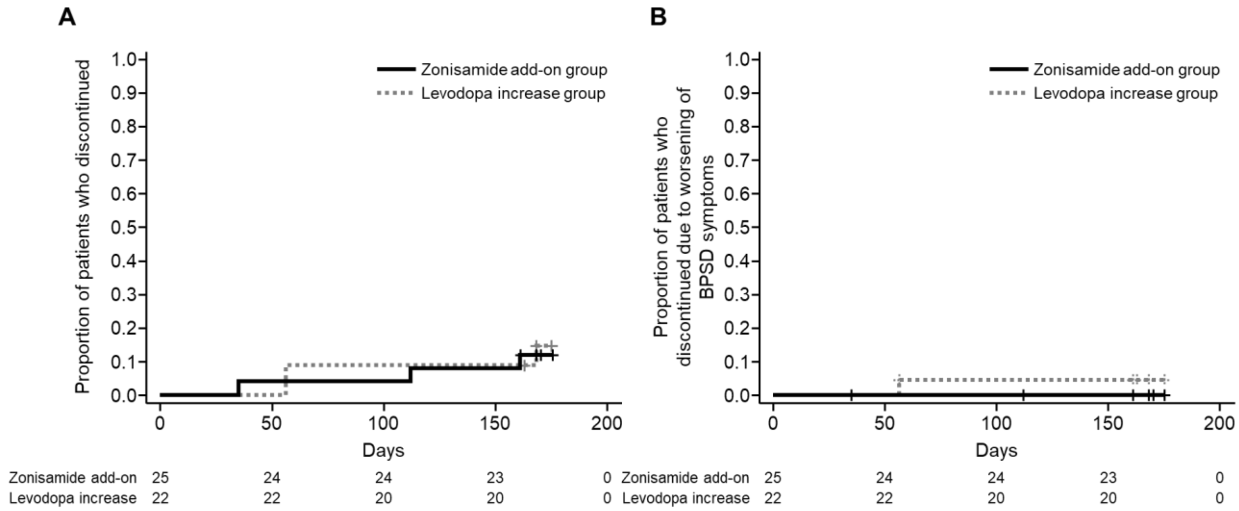

Log-rank $p = 0.361$ for discontinuation (zonisamide add-on group versus levodopa increase group)
Log-rank $p = 0.862$ for discontinuation for worsening BPSD symptoms (zonisamide add-on group versus levodopa increase group)
BPSD, behavioral and psychological symptoms of dementia
